# Supplementary material for: Decreased Consumption of Added Fructose Reduces Waist Circumference and Blood Glucose Concentration in Patients with Overweight and Obesity. The DISFRUTE Study: A Randomised Trial in Primary Care
Source: Nutrients. 2020 Apr 19;12(4):1149. doi: 10.3390/nu12041149 (PMC7231003; doi:10.3390/nu12041149)
Supplement: Supplementary file 1 [file nutrients-12-01149-s001.zip › supplementary files_Nutrients_711442/Table S1. Sources of nutrients and foods or dishes which were considered to contain added sugar.pdf]

FOOD	Ref. code	**Mataix reference**	**Name in food database or average value NOTES**
NON-WHOLE WHEAT BREAD (SANDWICH LOAF) (slice = 20 g)	c1	71	BIMBO DOS brand sandwich loaf
WHOLE WHEAT BREAD (SANDWICH LOAF) (slice = 20 g)	c2	74	
NON-WHOLE WHEAT BAGUETTE (grams)	c3	68	White bread
WHOLE WHEAT BAGUETTE (grams)	c4	78	Whole wheat bread
GOFIO (tablespoon = 20 g)	c5	46, 47	Average: wheat-corn
CEREALS WITH ADDED SUGAR (large spoonful = 30 g)	c6	30, 27, 31, 32, 63, 87, 89	Average: corn flakes with sugar (Kellogs), corn flakes with sugar (Pascual), puffed rice with honey (Pascual), toasted corn with honey and peanuts (Kellogs), puffed corn with honey, puffed wheat with honey, puffed wheat with sugar
CEREALS WITHOUT ADDED SUGAR (large spoonful = 30 g)	c7	13, 14, 28, 29, 33	Average: toasted puffed rice (Pascual), toasted puffed rice (Kellosgs), toasted corn flakes (Kellogs), toasted corn (Pascual), rice and wheat flakes (Special K)
Fried potatoes (grams)	c8		30 mL olive oil per 100 g. Homemade
Potatoes in stew (serving = 60 g)	c9	221	Boiled potato, per edible portion
TOAST (WHITE AND WHOLE WHEAT BREAD) (slice = 11 g)	c10		Ingredients from label (Hacendado brand)
PORK, SPARE RIBS, EXCLUDING PRESERVED MEATS IN CASING	c11	582, 584, 586, 594	Chop, ribs, loin, tenderloin
BEEF, VEAL g	c12	605, 614, 618	Lean beef, beef tenderloin
RABBIT, HARE g	c13	624	Rabbit
CHICKEN, POULTRY g	c14	629, 630, 631, 636, 637	Chicken, duck, turkey dark meat, chicken dark meat, chicken breast

CURED SERRANO HAM (slice = 30 g), (half a thin slice)	c15	650	
COOKED HAM, LOW-MOISTURE HIGH PROTEIN and REGULAR (slice= 20 g)	c16	649	Cooked ham
TURKEY BREAST (slice = 20 g)	c17	661	Turkey breast
FRESH LIVER (ORGAN MEAT) (grams) (similar to meatballs)	c18	585, 599, 609, 616, 635	Pork, lamb, beef, chicken liver
LAMB (grams)	c19	596, 601	Lamb chops and leg
EGGS (1 = 50 g)	c20	756	Chicken eggs
HAMBURGERS (WITH BUN), 1 = 185 g	c21	80, 1028, 1021, 1015	60 g bun, 10 g mustard, 15 g ketchup
HOT DOGS (WITH BUN), 1 = 120 g	c22	664, 80, 1021, 1015	45 g bun, 50 g hot dog, 10 g mustard, 15 g ketchup
WHITE FISH (COMMON DENTEX, PARROTFISH, SPLENDID ALFONSINO, BLACKSPOT SEABREAM, WRECKFISH) (grams)	c23	668,; 687, 689, 693, 696, 697, 711, 714	Inland grouper, gilt-head seabream, four-spot megrim, common sole, hake, grouper
BLUE FISH (TUNA, SARDINE, MACKEREL, RUBBERLIP GRUNT, MOROCCAN WHITE SEABREAM, BLACK SEABREAM) (grams) 5	c24	673, 678, 692, 710, 681	Tuna (albacore, skipjack tuna), chub mackerel, white trevally, horse mackerel, sardine
OCTOPUS, CUTTLEFISH, SQUID (grams)	c25	716, 727	Squid, octopus
SHELLFISH (LIMPET, SHRIMP, CRAB) (grams)	c26	715, 717, 719, 721, 722, 724	Clams (grooved carpet clam, striped venus clam), cockels, crab, velvet crab, spider crab, shrimp and popcorn shrimp, lobster and spiny lobster, mussels
SALTED OR DRIED FISH (grams)	c27	732, 733, 737, 738, 748	Smoked herring, salted herring, smoked cod, salted cod, smoked salmon
FRESH SAUSAGE (grams)	c28	663, 664, 665	Fresh sausage, frankfurter, Vienna sausage
ORGAN MEATS (TRIPE, KIDNEYS) (grams)	c29	583, 591, 597, 602, 603, 604, 607, 612, 613	Pork, lamb and beef tripe, kidneys, heart, brains

PORK BELLY, BACON, CHITTERLINGS (slice = 18 g)	c30	595, 638, 642	Pork belly, bacon, chitterlings
SPREADABLE CANARY ISLANDS SAUSAGE (1 = 40 g)	c31	667	Sobrasada (soft cured sausage)
CURED MEATS IN CASINGS (slice = 20 g)	c32	643, 644, 645, 648, 653, 656, 662	Pork bologna, turkey bologna, sausage, thin sausage (fuet), cured pork tenderloin, mortadella, salami
SATURN PEACH (small = 65 g, medium = 100 g, large = 135 g)	c33		BEDCA database: for sugars, equivalent values for portions of peach (sacarose 7 g, fructose 1.6 g, glucose 1.6 g)
SALAD (grams)	c34	194, 217, 222, 226, 232, 236, 238, 239, 242, 768, 839	Ingredients: onion, lettuce, cucumber, pepper, raw tomato, carrot, black olives, avocado, olive oil, vinegar
APPLE (grams)	c35	269, 270, 271	Red, Golden, Granny Smith apple
PEAR (grams)	c36	280	Pear
BANANA (grams)	c37	282	Banana
ORANGE, TANGERINE, GRAPEFRUIT, LEMON (grams)	c38	265, 267, 276, 283	Lemon, tangerine, grapefruit, orange
PAPAYA (grams SIMILAR TO SLICE OF MELON)	c39	279	Papaya
GRAPES (1 HANDFUL OR 1 SMALL BOWL) (EQUIVALENT TO 200 grams) (small grapes = 6 g), (large grapes = 12 g)	c40	289, 290	White and dark grapes
FRESH JUICE (fresh, not packaged) (mL)	c41	299, 307	Fresh-squeezed lemon and orange juice
FRUIT JUICE (PACKAGED) (mL)	c42	296, 302, 304, 305	Multifruit juice (Kasfruit), apple (Kasfruit), peach and grape (Kasfruit), orange (commercial brand)
PRESERVED FRUIT (SYRUP) (grams)	c43	314, 320, 329	Fruit cocktail in syrup, peaches in syrup, pineapple in syrup
FRESH TOMATO (1 = 145 g)	c44	232	Tomato
AVOCADO (1 = approx 150 g)	c45	242	Avocado
OLIVES (1)	c46	238, 239	Black and green olives
RAW ONION (1 = approx 110 g)	c47	194	Raw onion
RAW PEPPER (RED OR GREEN) (1 = approx 120 g)	c48	226	Raw pepper

CUCUMBER (1 = approx 150 g)	c49	222	Cucumber
MANGO (1 = approx 230 g)	c50	268	Mango
KIWI (1 = approx 90 g)	c51	262	Kiwi
FRESH FIG (1 = approx 50 g)	c52a	259	Green and black figs
PRICKLY PEAR (1 = approx 60 g)	c52	260	Prickly pear
LETTUCE (grams)	c53	217	Lettuce
RAW OR PRESERVED GARLIC (CLOVE)	c54	179	Garlic
POWDERED MILD (DRINK) mL	c55	455	Powdered milk
GOAT MILK mL	c56	428	Goat milk
LIQUID WHOLE MILK mL	c57	445	Cow milk (whole)
LIQUID LOW-FAT MILK mL	c58	439	UHT low-fat milk
LIQUID HALF-FAT MILK mL (and DANACOL brand = 100 mL)	c59	461	UHT half-fat milk (including Danacol)
MILK WITH VEGETABLE FAT (DAIRY PRODUCTS) mL	c60	435, 438	Low-fat mild with vegetable fat (olive oil) (Lilac), low-fat milk with vegetable oils
CONDENSED MILK (teaspoon = 10 g)	c61	430	Condensed milk
SOY MILK (mL)	c62		Sugar content from label (Hacendado brand). Others Mataix reference
CHOCOLATE SOY MILK (mL)	c63		Sugar content from label (Hacendado brand)
PETIT SUISSE (MANUFACTURED) (serving = 60 g)	c64	486, 489, 490, 493	Low-fat petit suisse with fruit (Danone brand), with fruit and cereals (Danone), plain with sugar (Danone), other flavors (Danone)
FLAN, VANILLA PUDDING (MANUFACTURED) (SERVING = 140 g)	c65	410, 413, 476, 479	Egg flan (Danone brand); vanilla flan (Danone), chocolate pudding (Danone), vanilla pudding (Nestle brand)
FLAVORED MILKSHAKE (MANUFACTURED) (mL)	c66	367, 369, 373, 375	Cocoa milkshake (Puleva); low calorie chocolate (McDonalds), low-calorie

			vanilla (McDonalds), vanilla UHT (Puleva)
DIARY CREAM (mL)	c67	470, 473, 474	Cream, pasteurized cream (Puleva brand), UHT cream (Puleva)
FRESH WHITE (UNCURED) CHEESE (g)	c68	495, 506	White low-fat cheese, fresh low-fat cheese (Danone brand), Burgos cheese (Arias brand)
SEMICURED CHEESE (YELLOW OR WHITE) (g)	c69	519, 523	Semicured manchego cheese, semicured full-fat cheese (Puleva brand)
CURED CHEESE (YELLOW OR WHITE) (g)	c70	505, 517, 520	Emmenthal, cured manchego, Parmesan
BUTTER (teaspoon = 20 g)	c71	776	Salt-free butter
MARGARINE (teaspoon = 20 g)	c72	780	Margarine
OLIVE OIL (teaspoon = 5 g) (tablespoon = 10 g)	c73	768	Olive oil
OTHER VEGETABLE OILS (teaspoon = 5 g)	c73bis	762, 765, 767, 771	Peanut, sunflower, corn, soy
MAYONNAISE (teaspoon = 10 g)	c74	783	Commercial mayonnaise
KETCHUP AND OTHER PROCESSED TOMATO SAUCES (teaspoon = 10 g)	c75	1015, 1026, 1027	Ketchup, uncooked strained tomato (Solis brand), fried tomato sauce (Orlando brand)
DRIED NUTS (PEANUTS, ALMONDS, WALNUTS) (tablespoon = 60 g or 1 handful)	c76	348, 351, 352, 353, 356	Almonds, hazelnuts, peanuts, chestnuts, walnuts
MOJO PICON SAUCE, TABASCO SAUCE (teaspoon = 15 g)	c77	179, 196 225, 768, 839	Garlic, red chilli, black pepper, olive oil, vinegar
MUSTARD (teaspoon = 10 g)	c78	1016	Mustard
SUGAR (g)	c79	131	Sugar
HONEY (teaspoon = 10 g)	c80	167	Honey
ICE CREAM (serving = 80 g)	c81	418	Ice cream, Mataix (proportion of carbohydrates: Finnish Institute: food no. 663: 69.3% sacarose and 30.7%

			lactose)
POPSICLE (1 =70 g) or SHERBET	c82		Finnish Institute: Food no. 31991 Sherbet (Carte D'or brand)
PLAIN WHOLE WHEAT FLOUR /MARÍA Brand COOKIES (1 = 6 g)	c83		Ingredients from label (Hacendado brand)
TRADITIONAL YELLOW CAKE (1 slice)	c84	55, 131, 444 , 776, 837	Wheat flour, sugar, whole milk, eggs, butter, leavening agent. Homemade
UNFILLED COOKIES CREMA (MARÍA brand) Number of cookies	c85	118	María brand cookies
FILLED COOKIES, including chocolate filling	c86	113, 117, 120	Chocolate cookies, Chocolate digestive biscuits, cocoa cream-filled ladyfingers (Dulcesol brand)
FILTERED COFFEE (DRIP COFFEE) (small cup)	c87		Expresso coffee
EXPRESSO COFFEE (home or cafés/restaurants) (mL)	c88	796	
DECAFFINATED COFFEE (mL)	c89	796	
INSTANT POWDERED CHOCOLATE DRINK (CONTAINING ADDED SUGAR (ColaCao, teaspoon = 5 g)	c90	793, 792	Powdered cocoa with sugar, ingredients from label (ColaCao brand), soluble cocoa (Nesquik brand)
CHOCOLATE (BARS, SQUARES, INDIVIDUAL CHOCOLATES) (small bar = 40 g)	c91	135, 136, 141, 144, 161, 162	Individual chocolates (Nestle brand), dark chocolate, white chocolate, extra fine milk chocolate with almonds (Nestle), extra fine dark chocolate with almonds (Nestle)
QUINCE OR GUAYABA QUINCE (slice = 15 g)	c92	321	Quince and fruit pastes
MARMALADE (spoonful = 12 g)	c93	322, 324,3 27	Apricot, plum, orange marmalade
HARD CANDIES, OTHER CANDIES (1 = 5 g)	c94	138, 14	Soft candies and chewing gum with sugar, including pacifier-shaped and round
DRIED FIGS (1 = 17 g)	c95	333	Dried figs
RAISINS (large spoonful)	c95bis	335	Raisins

WATERMELON (slice)	c96	285	Watermelon
MELON (slice)	c97	273	Melon
STRAWBERRIES (1)	c98	254	Strawberries
Cherries (1)	c99	246	Cherries
WATERCRESS (large spoonful)	c100	186	Watercress
HOT PEPPERS (WHOLE, NOT FOR MOJO SAUCE) (1)	c101	196, 197	Red chilli, green chilli
RICE (prepared in any manner) (grams)	c102	8, 9, 10, 11	Uncooked brown rice, boiled brown rice, uncooked milled rice, boiled milled rice
COOKED VEGETABLE (VEGETABLE BEAN, CARROT, ZUCCHINI SQUASH, CABBAGE, ROUND ZUCCHINI, PUMPKIN, COLIFLOWER) (grams)	c103	190, 192, 204, 206, 216, 237	Boiled zucchini squash, vegetable beans, pumpkin, carrots, cabbage, coliflower
CORN CAKES (1)	c104		Ingredients from label (Hacendado brand). Sugar assumed to be sacarose. Fat assumed to be 50% mono- and 50% polyunsaturated, saturated fats (according to label) subtracted from total fats
WHOLE GRAIN CEREALS (large spoonful = 30 g)	c105	22,66	All Bran (Kellogs brand), wheat bran (Hacendado brand), muesli (Kellogs)
CHOCOLATE-FILLED CEREALS (large spoonful = 30 g)	c106		Chocolate-filled cereal (Hacendado brand)
TEA (mL)	c107	826	Tea
SOFT DRINKS (LIGHT) (mL)	c108	788	Carbonated soft drinks (light)
SOFT DRINKS (REGULAR), CARBONATED (mL)	c109	822	Cola flavored soft drink
WINE, CAVA , CHAMPAGNE (mL)	c110	804, 833	Cava, table wine
BEER, CIDER (mL)	c111	805, 806,8 24	Beer, dark beer, cider
LIQUEURS AND SWEET WINES (mL)	c112	820, 821, 830, 831	Coffee liqueur, other liqueurs, sweet wine (Malaga), muscatel wine (glassful)

RUM AND COGNAC (mL)	c113	814, 823	Rum, cognac
BRANDY, WHISKY (mL)	c114	786, 835	Brandy, whisky
TAP WATER (mL)	c115		
BOTTLED WATER, CARBONATED (mL)	c116	785, 825	Mineral water (Perrier), soda water
BOTTLED WATER, STILL (mL)	c117	784	Mineral water
SOFT DRINKS, NON-CARBONATED (mL)	c117bis	789, 790	Isotonic drinks (Aquarius, Gatorade)
SALT (spoonful)	c118		Low-salt diet at www.fisterra.com. Dr María Carreira Miño
CANNED SARDINES OR TUNA (can = 85 g)	c119	734, 735, 749	Tuna in oil, tuna in brine, sardines in oil
ÑAME, SWEET POTATO (grams)	c120	183	Sweet potato
HERBAL TEA 1 (mL)	c121	826	
DRIED INSTANT SOUP (large spoonful = 100 g)	c122	989, 992, 994, 998, 999	Poultry with rice, chicken noodle, beef with vegetables, with small pasta (maravilla type)
PASTA COOKED AT HOME (MACARONI, SPAGHETTI, etc.) (grams) p. 20-21	c123	38, 41, 42, 59, 61	Whole wheat boiled spaghetti, regular boiled spaghetti with salt, regular boiled spaghetti without salt, boiled lasagna, boiled macaroni
STEW (large spoonful)	c124	62, 176, 179, 191, 194, 189, 215, 220, 223, 232, 236, 608, 768, 992	Corn, chard, garlic, pumpkin, onion, zucchini squash, green beans, potato, parsley, fresh tomato, carrot, beef ribs, olive oil
CANNED FOODS (STEWS, ETC.) (large spoonful)	c125	921, 923, 934, 937	Tripe with tomato and pepper (Tila brand), traditional chickpea stew (Litoral brand), Asturian white bean stew (fabada) (Litoral), lentils with sausage (Litoral)
PRECOOKED PIZZA (grams) 1 whole = 350 g, 1 slice = 44 g	c126	895, 899, 903	Tuna pizza, ham and cheese pizza, plain pizza (tomato sauce and cheese)

RANCHO CANARIO (large spoonful = 100 g	c127		Homemade, traditional recipe
TUNA AND VEGETABLE SALAD	c128		Homemade, traditional recipe
POTATO CHIPS (p. 92)	c129	1014	
CROISSANT (1 = 100 g)	c130	104	
CHOCOLATE-FILLED CROISSANT (1 = 100 g)	c131	105	
PLAIN MUFFIN (grams)	c132	119	
DONUT (PLAIN OR CHOCOLATE-COVERED (1 = 65 g)	c133	107, 108	Average: Plain and chocolate-covered donuts
Honey bee pollen (teaspoon = 10 g)	c134		Ingredients from label honey bee pollen (Hacendado brand) (sugar: apiculture journal 1993; no. 59 May)
VEGETABLE BEAN SALAD, grams	c135		Homemade, traditional recipe
Escaldón de gofio (porridge with stock and toasted flour) (serving = 150 g)	c136		Traditional recipe; content based on ingredients (1 serving = 150 g) not including mojo sauce
Potato omelette (grams)	c137		Homemade, traditional recipe
Chickpea stew (large spoonful = 100 g)	c138		Homemade, traditional recipe; also includes traditional dish from leftover stew (ropa vieja)
FRIED EGG (1 = 70 g)<br>(one)	c139		Homemade, traditional recipe
Roast leg of pork (slice = 25 g)	c140	590	
Cooked peas (grams)	c141		
Potato salad (serving = 120 g)	c142		Homemade, traditional recipe
Lentil stew (large spoonful = 100 g)	c143		Traditional recipe; content based on ingredients
Sweet buns, rolls, muffins, etc. (grams)	c144		Ensaimada = 37 g, valenciana = 30 g, apple tart = 74 g, sobao = 20 g, ladyfingers = 40 g

Flaky scroll pastry (plain, chocolate-covered, whole wheat) large = 115 g, small = 23 g)	c145		Ingredients from label (Eidetesa brand)
Tree tomato (1 = 30 g)	c146		
Hazelnut and chocolate spread (grams)	c147		Ingredients from label (Nocilla brand)
Popcorn (serving = 25 g)	c148		Ingredients from label (Auchan brand popcorn)
Sweetened fruit juice (mL)	c149		Ingredients from label, pear-pineapple juice (Juver and Libby´s brands)
LOW-FAT SWEETENED YOGURT	c150		Ingredients from label (Hacendado brand)
PLAIN YOGURT (SWEETENED, WITH OR WITHOUT FRUIT)	c151	558, 567, 578	Plain yogurt with sugar, with or without fruit (Danone brand)
PLAIN GREEK YOGURT	c152		Ingredients from label (Hacendado brand)
GOAT MILK YOGURT	c153		Ingredients from label (Romar brand, Mercadona)
SOY/LACTOSE-FREE YOGURT	c154		Ingredients from label (Hacendado and Kaiku brands, Mercadona)
Baked flat bread (arepa) (50 grams)	c155		Homemade, traditional recipe
Plums (1 = 60 g)	c156	129	
PLAIN OMELETTE: 1 egg 55 g, 2 eggs 110 g	c157		Homemade, traditional recipe
Peach, Apricot	c158	243, 272	
CROQUETTES (1 = 35 g)	c159	861	
Copa de Chocolate/nata de DANONE (1 = 150 g)	c160		Calculated from ingredients (Homemade)
Wheat flatbread for burritos (1 = 40 g)	c161		USDA: 18970, Tortillas, ready-to-bake or -fry, flour, shelf-stable
CREAM CHEESE (PHILADELPHIA brand)	c162		USDA: 01017, Cheese, cream

CHICKEN NUGGETS (1 = 26 g)	c163		Homemade, traditional recipe
SURIMI (1 = 18 g)	c164		Package
POTATO PURÉE, grams	c165	983	Mataix: Potato purée (Maggi brand). Sugar and starch content estimated from proportions in potato
FRESH PINEAPPLE WITH JUICE, grams	c166	329	
COMMON MUSHROOMS, OYSTER MUSHROOMS, grams	c167	195	
LEGUME BEANS, grams	c168	216	
COLESLAW, CARROT SALAD, BEET SALAD, grams	c169		Homemade, traditional recipe
FRIED FLAT BREAD (AREPA) (1 = 90 g)	c170		Homemade, traditional recipe
SWEET CORN (grams)	c171	62	
VINEGAR (mL)	c172		USDA (Mataix table for proportions of sugars and starch)
CACHAPA (thick crepe) (1)	c173		Homemade, traditional recipe
SMALL MEAT PIE (1)	c174		Homemade, traditional recipe
TEQUEÑO (fried bread stick) (1)	c175		Homemade, traditional recipe
INSTANT GELATIN (1 = 125 g)	c176		Ingredients from label (Royal brand)
SANCOCHADO (meat or fish, potato, vegetables, porridge) BANANA (1)	c177		Homemade, traditional recipe
COCONUT PASTRY (1 = 20 g)	c178		MERCADONA Brand, Ingredients from label
HALLACA (tamale) (1)	c179		Homemade, traditional recipe
GUAYABA (1)	c180	258	
Prickly pear (1)	c181	260	
EGGPLANT (AUBERGINE) (COOKED) grams	c182	185	
DATES (1 = 10 g)	c183	252	
PUCHERO CANARIO (Canary Islands stew: chickpeas, vegetables, meats, poultry) (serving = 300 g)	c184		Homemade, traditional recipe

Apple pastry (manufactured commercial brand) (1 = 65 g)	c185		Teisol internet site. Approximate sugar content based on apple, monosaturated and polyunsaturated fatty acids
CHEESE PUFFS (Risi brand, 1 package = 35 g)	c186		Label: sugar content estimated assuming cooked corn (Mataix)
(SAN JACOBO) BREADED FRIED HAM AND CHEESE ROLL (1 with oil = 102 g)	c187		University of Murcia. Findus brand San Jacobo
POLVORÓN (SHORTBREAD COOKIE) (40 grams)	c188		Homemade, traditional recipe
FRIED BATTER-COATED HAKE (grams) 1 HAKE FISH STICK (1 = 30 g)	c189		Pescanova brand, plus oil for frying
STEWED BEEF (large spoonful = 150 g, serving = 270 g)	c190		Homemade, traditional recipe
FRIED RICE WITH EGG, VEGETABLE AND MEAT OR SHRIMP (grams)	c191		Homemade, traditional recipe
FRIED BATTER-COATED SQUID (serving = 120 g)	c192		Homemade, traditional recipe
FRIED BATTER-COATED FISH (1 = 25 g)	c193		Homemade, traditional recipe
FRIED DOUGH (1 = 25 g)	c194		Homemade, traditional recipe
SPRING ROLL (1 = 89 g)	c195		USDA
PERSIMMON (grams)	c196	245	
LIQUID YOGURT (Actimel/DanActive probiotic) (1 = 100 mL)	c197	359	Mataix (see entry for sugars)
MERINGUE (1 = 8 g)	c198		Finnish Institute (homemade, traditional recipe)
LUPINES (grams)	c199		USDA: 16077, Lupines, ripe seeds, cooked, boiled, sugar = 0 (Ingredients from label, Mercadona brand; no other sources found)
MEDLAR (1 = approx 80 g)	c200	278	Mataix (sugar according to dietary proportion in www.dietas.net: glucose 26.6%; fructose 37.3%, sucrose 36.1%)

BEET (grams)	c201	230	
BLOOD SAUSAGE (grams)	c202	655	Mataix (carbohydrates = 3 g, assuming all are sucrose according to USDA: 07005, blood sausage)
SOY LECITHIN (grams)	c203		Delyplus brand (Mercadona) (sugar content = sucrose)
ALMOJÁBANA (cheese cake) (1 serving = 75 g)	c204		Homemade, traditional recipe
GAZPACHO (mL)	c205		Homemade, traditional recipe
ALMOGROTE (cheese and pepper spread) (mL)	c206		Homemade, traditional recipe
CHESTNUTS (1 = 10 g)	c207	166	
PATÉ (FOIE GRAS OR OTHER) (grams)	c208	658	Mataix (sugar content calculated from percentages in Finnish Institute table)
HARD ALMOND NOUGAT (1 piece = 40 g)	c209		Homemade, traditional recipe
APPLE COMPOTE (grams)	c210		Homemade, traditional recipe
PASSION FRUIT (grams)	c211		Danish database: no. 1055
FRUCTOSE (sugar)	c212		
SOY HAMBURGER	c213		USDA 16147
SUGARED ALMONDS (1 = 4 g)	c214		Homemade, traditional recipe
ENERGY GEL	c215		Ultra Gel 700 (Decathlon Brand)
PEAS WITH OTHER VEGETABLES, SAUSAGE AND EGG	c216		Homemade, traditional recipe
MORINGA (drumstick tree): dried leaves (grams)	c217		USDA
SESAME SEEDS (grams)	c218		USDA
TUNA LASAGNA (grams)	c219		Homemade, traditional recipe
MEAT LASAGNA (grams)	c220		Homemade, traditional recipe
VEGETABLE LASAGNA (grams)	c221		Homemade, traditional recipe
TRUCHA DE BATATA (yam, almond and sugar-filled pastry) (1 portion)	c222		Homemade, traditional recipe
CAKE OF ANY FLAVOR OR TYPE EXCEPT APPLE (grams)	c223		Homemade, traditional recipe

APPLE CAKE (grams)	c224		Homemade, traditional recipe
SPINACH-FILLED CANNELLONI (grams)	c225		Homemade, traditional recipe
TIRAMISU (grams)	c226		Homemade, traditional recipe
Flakey pastry (g)	c227		Homemade, traditional recipe
Candied pumpkin or squash (g)	c228		Homemade, traditional recipe
TRUCHA DE CALABAZA (candied, spiced pumpkin- or squash-filled pastry) (1 portion = 100 g)	c229		Homemade, traditional recipe
PAN DE LECHE (sweet roll) (1 portion = 40 g)	c230		Homemade, traditional recipe
Fried anisette-flavored donut (1 portion = 50 g)	c231		Homemade, traditional recipe
Meringue-topped cake (g)	c232		Homemade, traditional recipe

Sources for nutrient contents:
- Mataix (reference 15)
- Base de datos española de composición de alimentos (BEDCA): http://bedca.net/
- United States Department of Agriculture. Agricultural research service. (USDA): https://ndb.nal.usda.gov
- National Institute for Health and Welfare, Fineli (Finnish Institute): https://fineli.fi/fineli/en/index?
- Danish food composition data: http://frida.fooddata.dk/index.php?lang=en

Note about the names of fish species in this Annex:
The common name in Spanish as used locally in the Canary Islands was verified against the regional fisheries authority's official guides to species of commercial interest (http://pescamax.foroactivo.com/t720-peces-de-interes-pesquero-en-las-islas-canarias-por-www-jornadasdepesca-blogspot-com), and once the Latin name of the species was identified, the common name in English was obtained from Fishbase (http://www.fishbase.org/search.php).

These foods or dishes were considered that come from industry or with added sugar:

c6 c42 c43 c61 c64 c65 c66 c75 c79 c80 c81 c82 c83 c84 c85 c86 c90 c91 c93 c94 c104 c105 c106 c108 c109 c112 c130 c131 c132 c133 c134 c144 c145 c147 c148 c149 c150 c151 c152 c153 c154 c160 c176 c177 c178 c185 c186 c188 c197 c198 c209 c210 c212 c214 c215 c222 c223 c224 c226 c227 c228 c229 c230 c231 c232 .
